# Implications of Attrition in a Longitudinal Web-Based Survey: An Examination of College Students Participating in a Tobacco Use Study

**DOI:** 10.2196/publichealth.7424

**Published:** 2017-10-16

**Authors:** Bennett McDonald, Regine Haardoerfer, Michael Windle, Michael Goodman, Carla Berg

**Affiliations:** ^1^ Emory University Atlanta, GA United States

**Keywords:** young adults, risk factors, tobacco use, methods, surveys and questionnaires

## Abstract

**Background:**

Web-based survey research has several benefits, including low cost and burden, as well as high use of the Internet, particularly among young adults. In the context of longitudinal studies, attrition raises concerns regarding the validity of data, given the potential associations with individual and institutional characteristics, or the focal area of study (eg, cigarette use).

**Objectives:**

The objective of this study was to compare baseline characteristics of nonresponders versus responders in a sample of young adult college students in a Web-based longitudinal study regarding tobacco use.

**Methods:**

We conducted a secondary data analysis of 3189 college students from seven Georgia colleges and universities in a 2-year longitudinal study. We examined baseline tobacco use, as well as individual- and institutional-level factors, as predictors of attrition between wave 1 (October and November 2014) and wave 2 (February and March 2015) using multilevel modeling. **Results:** A total 13.14% (419/3189) participants were lost to follow-up at wave 2. Predictors of nonresponse were similar in the models examining individual-level factors and institutional-level factors only and included being black versus white (odds ratio [OR] 1.74, CI 1.23-2.46); being male versus female (OR 1.41, CI 1.10-1.79); seeking a bachelor’s degree versus advanced degree (OR 1.41, CI 1.09-1.83); not residing on campus (OR 0.62, CI 0.46-0.84); past 30-day tobacco use (OR 1.41, CI 1.10-1.78); attending a nonprivate college (OR 0.48, CI 0.33-0.71); and attending a college with ≤10,000 students (OR 0.56, CI 0.43-0.73).

**Conclusions:**

Future longitudinal studies should assess predictors of attrition to examine how survey topic and other individual and institutional factors might influence the response to allow for correction of selection bias.

## Introduction

Rapid development of new information technology tools has revolutionized survey implementation. In parallel with this innovation, however, researchers have noticed a universal and consistent decline in survey participation. The rate of nonresponse in survey research has increased within the past decade when compared with the latter half of the 20th century [1,2]. The inability to recruit and retain study participants within longitudinal studies poses potential threats to the validity of population-based studies when the participation itself is related to survey or other unobserved variables [3-5]. Further, investigations of the potential threats to validity (ie, biases) due to nonparticipation are often insufficiently explored, particularly, in studies of substance use by young adults [6-9].

Although avoiding bias is desirable in public health research, researchers conducting longitudinal studies are often forced to contend with unit nonresponse. Unit nonresponse, in the context of this analysis, refers to a failure to retain participants in subsequent waves of a study after some initial participation and is often viewed as difficult to interpret and account for during analysis. Although loss to follow-up does not always result in biased estimates, problems arise when the nature of the nonresponse becomes systematic with regard to individual or institutional factors. Additionally, having a high-retention rate between waves does not guarantee unbiased estimation if this systematic attrition exists [2,4,9].

Previous longitudinal studies of tobacco and other substance use in student and young adult populations have encountered varying levels of attention to missing data. Methods for managing attrition have included comparing characteristics of participants and nonparticipants [10-13], comparing early versus late respondents in terms of recruitment period [14], reporting conservative baseline estimates [15], or descriptive analysis of those retained [16]. Mixed results have been reported from studies where correlates were explored.

Topic of survey and substance use may be two factors contributing to attrition. For example, one study of hookah use by young adults transitioning from high school to college found that those who completed a 1-month follow-up survey were more likely to be lifetime hookah nonusers and lifetime smokeless tobacco nonusers; however, current alcohol use and current cigarette use were unrelated to attrition [17]. Similarly, prior research found that those who participated at the final wave of data collection were less likely to have smoked the month before the wave 1 [18]. Other tobacco studies in this population, however, found no associations with substance use [13]. Given the mixed findings regarding the presence or absence of previous tobacco or other substance use and attrition, examining correlates of attrition and attempting to understand reasons for attrition is a vital step in the research process. For example, if a study is primarily concerned with tobacco use [19], the literature suggests that those dropping out might do so because of a lack of topic salience or relevancy. Conversely, it is also possible that those who use tobacco might be dropping out at a higher rate because of perceived stigma related to smoking or other tobacco use [20].

Very few studies have examined predictors of study retention at the higher education and institutional level (eg, school level for individual student attrition). In terms of sociodemographics, prior research in adolescents and young adults has found greater attrition and recruitment nonresponse among men [21-25], non-whites [17,22], and those whose parents reported lower education levels [9,22]. Few studies have investigated the association between school status (eg, public vs private) and attrition rates. Although, one study found that students recruited from public schools or those located in urban areas showed higher odds of nonresponse [26]. Additionally, institutions with larger student population size have exhibited lower levels of survey participation [26]. Another factor worth exploring is the place of residence (on campus vs elsewhere); smoking status may be different because of smoke-free policies and because of the potential of greater exposure to other students participating in the study both of which may influence response. Cross-sectional research has suggested that those who live on campus are more likely to participate [24]. Although attention has been given to the relationship between survey recruitment and institutional factors, further efforts need to be taken to understand area-level predictors of longitudinal survey retention.

In this study, we conducted a secondary data analysis to estimate the association between baseline (wave 1) 30-day substance use, specifically tobacco use, and wave 2 attrition using a sample of young adult college students participating in a longitudinal Web-based survey study of tobacco use. In addition, we explored individual (eg, sociodemographics, academic variables, and place of residence) and institutional factors (eg, urban or rural location, type of college or university, and school size) associated with wave 2 attrition, using a multilevel mixed effects modeling approach.

Drawing from the literature, we hypothesized that attrition will be higher for non-whites, men, those whose parents reported lower education levels, those seeking lower academic degrees, and those residing off campus. Second, we hypothesized that attrition will be higher for those at urban schools versus rural schools and those at schools with larger student populations compared with those with smaller student populations. Of note, given the contradictory findings in the literature regarding substance use and attrition, we do not have a specific hypothesis but will explore this potential predictor.

## Methods

### Study Design and Participants

Project DECOY—Documenting Experiences with Cigarettes and Other Tobacco in Young Adults—is a quantitative longitudinal assessment of tobacco use predictors in Georgia college students [19]. Our overall study and reporting approaches were guided by the Checklist for Reporting Results of Internet E-Surveys (CHERRIES) guidelines [27]. This study was approved by the institutional review boards of the Emory University and ICF, International, as well as those of the participating colleges.

The study was initiated in 2014. Data were collected from seven Georgia colleges, including two public schools, two private schools, two community colleges and technical colleges, and one historically black university. Surveys were administered every 4 months across six waves of data collection during spring, summer, and fall.

Eligible participants were aged between 18 and 25 years and were able to read English. A list of students was obtained from each institution’s office of the registrar. Using these lists as the sampling frame, 3000 randomly selected students from each one of the private school and two public schools were invited to participate. The remaining institutions contained fewer than 3000 students, and the invitations were emailed to all eligible participants. The invitation emails described the study (longitudinal study with six assessments over 2 years) and the incentives for participating. If potential participants were interested, they clicked on a link embedded in the email, which launched them to the consent form. After reading the consent form, they had the option to consent by clicking a link, which then launched the baseline (wave 1) survey. Recruitment at each school was closed after recruitment goals at each school were reached. Response rates ranged from 11.99% (1872/15,607) to 59.40% (9270/15,607), with an overall response rate of 22.90% (3574/15,607). A week after completion of the baseline survey, participants were asked to confirm their participation in the study via an emailed link and were provided their first gift card (US $30). The response rate after confirmation was 95.64% (3418/3574).

Several techniques were used to increase the retention of the participants during follow-up [19]. In brief, social media contacts (ie, Facebook and Twitter), in addition to primary and alternate email addresses, were obtained to increase probability of reaching participants. An option to provide contact information of relatives and others likely to know the whereabouts of the participant was given. Additionally, small gifts (eg, key chains) were provided, as well as access to Project DECOY social media was employed to enhance engagement with the participants. Before launching each wave of assessment, updates and reminders regarding survey procedures were provided through the DECOY Twitter account and Facebook page, and text messages were sent to the participants 1 month before the survey’s administration via Trumpia (DoCircle, Inc, Anaheim, California) to remind participants of the survey and to allow them to update their email address. Once each wave of assessment was launched, up to 5 email reminders within a 4-week period were sent before the opportunity to participate in that wave was closed. To further encourage participation, survey incentives were provided on a gradual schedule (US $30 for the first 2 waves, US $40 for the second 2 waves, and US $50 for the final 2 waves). If participants completed all 6 surveys, they received an additional US $100 (for a total of US $340). If participants did not complete 1 wave of assessment, they were eligible for the subsequent waves of assessment, regardless.

The secondary analysis examined predictors of baseline (wave 1) participants completing the wave 2 (ie, 4-month follow-up) assessment. After assessing refusal or missing data for variables included in the final model, our analytic sample comprised 93.30% (3189/3418) of the confirmed participants. A total of 13.14% (419/3189) of the participants were lost to follow-up at wave 2.

### Measures

The baseline DECOY survey was developed by Emory University, programmed by ICF, International and pilot-tested by both Emory University and ICF, International to ensure functionality of the programming and survey content. The baseline assessment was administered via the Web using a closed survey (ie, only invited participants could access the survey), which involved each user being assigned a unique link to monitor that individual’s response (ie, only one response allowed). The survey took between 30 and 45 mins to complete. Measures included a range of variables, such as sociodemographics, general health information, psychosocial characteristics, and substance use, all of which were presented in the same order for all participants. Certain skip patterns were applied (ie, those not reporting past 30-day tobacco use skipped the section regarding types of products used, use frequency, readiness to quit, etc). Participants were required to respond to each question, with particularly sensitive questions (eg, illegal substance use) having a response option of *efuse*. Participants were not allowed to return to prior screens of the survey once they had moved on in order to prevent participants from retroactively changing answers to move past sections involved in skip patterns. However, each page included study staff contact information to correct errors. All data were automatically recorded via ICF, International’s software and were stored in secure servers at ICF, International transferred to Emory University using a secure portal, and then stored in secured servers at Emory University.

#### Substance Use

To assess tobacco use, we first asked participants whether they had used a range of tobacco products (cigarettes; e-cigarettes; hookah; flavored little cigars or cigarillos; and chewing tobacco, snuff, or dip, snus–collectively called smokeless tobacco) in their lifetime at wave 1 using standard items from the Centers for Disease Control and Prevention National Adult Tobacco Survey. Those indicating lifetime use were then asked to report the number of days they used the respective tobacco products in the past 30 days. A similar approach was taken for assessing alcohol use.

#### Individual-Level Factors

Several individual-level characteristics were assessed, including age; race and ethnicity (non-Hispanic white, non-Hispanic black, other); sex; highest level of parental education; highest level of degree sought; and place of residence (on campus vs other).

#### Institutional-Level Factors

The following three types of institutional factors were examined: (1) rural versus urban status of the area in which the institution resided (based on census classification); (2) type of school (private, public, community or technical, historically black); and (3) student population (< vs >10,000 based on distribution of population sizes). On the basis of our preliminary analyses, the type of school was operationalized as private school versus other given differential nonresponse rates.

### Data Analysis

First, bivariate associations between each predictor and nonresponse at wave 2 were assessed. *t* tests and chi-square (or Fisher exact test) tests were used for continuous and categorical variables, respectively, comparing baseline data for responders and nonresponders. The Wilcoxon rank-sum test was used for variables with distributions, which were non-normal.

A generalized linear mixed model containing all relevant individual- and institutional-level variables was used to determine predictors of nonresponse at wave 2. Results were expressed as adjusted OR with the corresponding 95% CI. An unconditional model with no variables entered was first used to estimate the intracluster correlation coefficient (ICC), which describes the variability in nonresponse at wave 2 because of the nesting of students within the institution. Students attending the same institution are assumed to be more similar because of the characteristics of that institution, and this variability due to institution may also be explored in addition to the effect of individual characteristics. Models containing only individual-level variables, only institutional-level variables, and both individual and institutional variables were then constructed. Individual characteristics were entered into the model based on a priori considerations. Model fit statistics were calculated and likelihood ratio tests were used to compare the change in deviance of nested models. Lastly, the reduction in level-2 (school level) variance compared with the unconditional model was calculated for each subsequent model. All analyses were conducted in SAS Institute’s SAS version 9.4 (Cary, North Carolina, USA), and alpha was set at .05.

## Results

Table 1 provides descriptive characteristics and bivariate analyses comparing those who responded to the wave 2 assessments versus the nonresponders to the wave 2 assessment. Note that, at baseline (wave 1), over a quarter (949/3189, 29.76%) of the participants indicated that they had used at least one tobacco product within the last 30 days, and over half reported past 30-day alcohol use (2019/3189, 63.31%).

Total 13.14% (419/3189) of the participants were lost to follow-up at wave 2. Baseline (wave 1) predictors of being lost to follow-up at wave 2 included being black (*P*<.001); having parents’ with an advanced degree (*P*<.001); seeking an associate’s or bachelor’s degree (*P*<.001); not residing on campus (*P*<.001); past 30-day tobacco use (*P*<.001), specifically cigarette (*P*<.001), hookah (*P*=.024), and little cigar and cigarillo (*P*<.001) use; and attending an urban college, a nonprivate college, and college with smaller student populations (ie, ≤10,000 students; *P*<.001).

Results from the model building process are found in Tables 2 and 3. First, the unconditional model containing no predictors was fit. Covariance parameter estimates indicated an ICC of 10.1%, indicating that 10.1% of the variability in nonresponse was due to between-school-level characteristics. Model selection criteria and likelihood ratio tests using deviance statistics indicated that model fit increased significantly when comparing the model containing individual predictors only (*P*<.001) and institutional predictors only (*P*<.001) to the unconditional model. The full model containing all predictors was tested against the individual level model and had significantly better fit (*P*<.001). The Akaike information criterion (AIC) and the Bayesian information criterion (BIC) fit statistics were consistent with these findings. Given the optimal fit of the full model, the parameter estimates of the full model are interpreted. Predictors of nonresponse were similar in the models examining individual-level factors and institutional-level factors only, respectively, and included being black (vs white; OR 1.74, CI 1.23-2.46); being male (vs female; OR 1.41, CI 1.10-1.79); seeking a bachelor’s degree (vs advanced degree; OR 1.41, CI 1.09-1.83); not residing on campus (vs residing on campus; OR 0.62, CI 0.46-0.84); past 30-day tobacco use (vs nonuse; OR 1.41, CI 1.10-1.78); attending a nonprivate college (vs a private college; OR 0.48, CI 0.33-0.71); and attending a college with <10,000 students (vs a college with >10,000 students; OR 0.56, CI 0.43-0.73).

**Table 1:** Descriptive characteristics and bivariate associations of institutional and individual factors associated with nonresponse at wave 2 among young adults in a longitudinal cohort study.

Variables			Overall sample (N=3189)	Responders (n=2770)	Nonresponders (n=419)	*P* value^a^
			n (%)	n (%)	n (%)	
**Individual level**						
	Age (mean, SD)		20.54 (1.96)	20.53 (1.93)	20.60 (2.13)	.35
	**Race**					<.001
		White	2010 (63.03)	1795 (64.80)	215 (51.31)	
		Black	793 (24.87)	634 (22.89)	159 (37.95)	
		Other	386 (12.10)	341 (12.31)	45 (10.74)	
	**Sex**					.94
		Female	2053 (64.38)	1784 (64.40)	269 (64.20)	
		Male	1136 (35.62)	986 (35.60)	150 (35.80)	
	**Parental education**					<.001
		No college degree	864 (27.09)	781 (28.19)	83 (19.81)	
		Bachelor's degree	1098 (34.43)	962 (34.73)	136 (32.46)	
		Advanced degree	1227 (38.48)	1027 (37.08)	200 (47.73)	
	**Degree sought**					<.001
		≤Associate’s degree	226 (7.09)	182 (6.57)	44 (10.50)	
		Bachelor’s degree	652 (20.45)	537 (19.39)	115 (27.45)	
		Advanced degree	2311 (72.47)	2051 (74.04)	260 (62.05)	
	Reside on campus		1397 (43.81)	1266 (45.70)	131 (31.26)	<.001
	Past 30-day tobacco use		949 (29.76)	828 (29.89)	184 (43.91)	<.001
	**Past 30-day use by product**					
		Cigarettes	421 (13.20)	344 (12.42)	77 (18.38)	<.001
		E-cigarettes	345 (10.82)	290 (10.47)	55 (13.13)	.10
		Hookah	419 (13.14)	329 (11.88)	74 (17.66)	.02
		Little cigars/cigarillos	363 (11.38)	289 (9.35)	74 (17.66)	<.001
		Smokeless tobacco	114 (3.57)	97 (3.50)	17 (4.06)	.57
		Past 30-day alcohol use	2019 (63.31)	1743 (62.92)	276 (65.87)	.24
**Institutional level**						
	Urban school (vs rural)		1218 (38.19)	1003 (36.21)	215 (51.31)	<.001
	Private school (vs other)		1236 (38.76)	1155 (41.70)	81 (19.33)	<.001
	Student population >10,000		1412 (44.28)	1271 (45.88)	141 (33.65)	<.001

^a^*P* value comparing responders and nonresponders using Student *t* test for continuous variables and chi-squared for categorical variables.

^b^Those who answered “don’t know,” “refuse,” or were in a respondent group with very small cell size for one or multiple covariates (N=229) were coded missing for the analytic sample. Missing values occurred for Parental education (N=48), Race (N=45), Degree sought (N=158), Sex (N=4 reporting “other”), and School (N=74).

**Table 2:** Results from a multilevel model assessing institutional and individual factors associated with nonresponse at wave 2 among young adults in a longitudinal cohort study (n=3189).

Variables			Unconditional model	Individual level	Institutional level	Full model
			OR^a^ (95% CI)	OR^a^ (95% CI)	OR^a^ (95% CI)	OR^a^ (95% CI)
**Individual level**						
	Age			0.94 (0.89-1.00)		0.95 (0.89-1.01)
	**Race**					
		White		ref		ref
		Black		1.57^b^ (1.14-2.16)		1.74^b^ (1.23-2.46)
		Other		1.30 (0.90-1.87)		1.43 (0.99-2.05)
	**Sex**					
		Female		ref		ref
		Male		1.36^b^ (1.07-1.74)		1.41^b^ (1.10-1.79)
	**Parental education**					
		No college degree		1.02 (0.75-1.39)		0.97 (0.72-1.32)
		Bachelor’s degree		1.01 (0.74-1.38)		0.97 (0.71-1.31)
		Advanced degree		ref		ref
	**Degree sought**					
		<Associate’s degree		1.05 (0.69-1.60)		1.01 (0.68-1.52)
		Bachelor’s degree		1.46^b^ (1.22-1.90)		1.41^b^ (1.09-1.83)
		Advanced degree		ref		ref
	Reside on campus			0.62^b^ (0.46-0.85)		0.62^b^ (0.46-0.84)
	Past 30-day tobacco use			1.42^b^ (1.12-1.80)		1.41^b^ (1.10-1.78)
	Past 30-day alcohol use			1.14 (0.89-1.47)		1.14 (0.89-1.45)
**Institutional level**						
	Urban school (vs rural)				1.14 (0.74-1.79)	1.05 (0.75-1.47)
	Private school (vs other)				0.37^b^ (0.22-0.60)	0.48^b^ (0.33-0.71)
	Student population >10,000				0.59^b^ (0.41-0.86)	0.56^b^ (0.43-0.73)

^a^OR: odds ratio.

^b^*P*<.05.

**Table 3:** Model fit from a multilevel model assessing institutional and individual factors associated with nonresponse at wave 2 among young adults in a longitudinal cohort study (n=3189).

Model fit criteria	Unconditional model	Individual level	Institutional level	Full model
τ_00_	0.3694	0.2112	0.0417	0.0014
Reduction in τ_00_	ref	51.0%	89.6%	99.4%
Deviance	2547.55	2336.54	2534.65	2321.32
Akaike information criterion	2551.55	2362.54	2544.65	2353.32
Bayesian information criterion	2551.44	2361.84	2544.38	2352.46
χ^2^ (degrees of freedom = 14)	ref	211.0^a^	12.90^a^	15.2

^a^*P*<.05.

## Discussion

### Principal Findings

This study investigated individual- and institutional-level predictors of dropouts from a longitudinal survey in a diverse sample of students enrolled in universities of various types. Previous literature showed that dropping out was related to a few key factors relevant to this study, including survey topic salience [13,17-20], behaviors related to the survey topic [13,17-20], participant sociodemographics [9,17,21-25], and institutional factors [24-26].

The results of the multilevel model indicated that 30-day tobacco use predicted nonresponse at wave 2. This finding is consistent with survey attrition research conducted with young adults in the general population [18] although the subject matter of these studies was not primarily concerned with tobacco use, or they studied unique populations such as young adult military personnel. It is unique in its relevancy to college students participating in a study primarily concerned with tobacco use, and it rejects the idea that student’s dropping out is largely because of a lack of interest or relevance with regard to the survey topic. Instead, attrition could be related to feelings of stigmatization as a tobacco user in a less socially acceptable setting or some other unmeasured factor; that is, young adults may attempt to avoid cognitive dissonance related to either not reporting their behaviors because of stigmatization or related to reporting behaviors that are stigmatized. It is also unique in its consideration of contextual- and institutional-level factors such as school size, school type, and urban rural status.

At the individual level, many of our findings were consistent with previous literature. For instance, higher odds of nonresponse were seen in blacks and in men; similar results that have been replicated in many different samples of college students [17,23-25]. We also found that, compared with those seeking an advanced degree, those seeking a bachelor’s degree–but not those seeking an associate’s degrees–were more likely to be lost at follow-up, indicating a curvilinear relationship. This finding warrants further examination but may be related to parental education and reflects financial motivation because of potentially being from a lower socioeconomic background (which was significant in the bivariate but not multivariate analyses); this is particularly compelling, given that the degree sought and parental education are correlated in this sample. Additionally, those living off campus were more likely to be nonresponders at wave 2, which aligned with our hypotheses.

Institutional variables also accounted for varying levels of nonresponse. For example, private schools exhibited lower odds of nonresponse compared with public schools. In our sample, private schools had smaller student populations, which were predictive of responding at wave 2, which is consistent with prior findings. School’s urban or rural status was not associated with nonresponse. This, however, could be because of the relatively small number of schools and should be investigated with a more representative sample.

### Study Strengths and Limitations

In terms of strengths, selecting from a diverse group of schools and participants provided a heterogeneous sample relative to many other studies of student populations, including students of different races and ethnicities, urban and rural status, and socioeconomic status. Furthermore, the use of individual- and institutional-level variables has been rare in studies of cross-sectional and longitudinal nonresponse, particularly, in studies of students’ tobacco and substance use, and our analysis of both levels is important for building a greater understanding regarding the factors that are most impactful when college students are deciding to continue participating in a tobacco use study.

However, a small number of schools were used to predict school characteristics associated with nonresponse, given that this was a secondary analysis of data. Although simulation studies have shown that inferences can still be drawn with a low number of area-level units [28], caution should be exercised in drawing conclusions from these results. Second, certain covariates utilized measurement scales available for a secondary data analysis, such as parental education as a proxy for socioeconomic status; alternative methods should be used to explore these associations further. Additionally, although institutional variables were included, the nature of community or technical colleges may account for unexplained variance (eg, socioeconomic status and residential differences); this could be explored in future studies. Additionally, other factors such as college major, jobs, and extracurricular activities may have been relevant; however, these factors are difficult variables to operationalize given their variety (and instability). Lastly, this study analyzed dropout between two subsequent waves of a longitudinal study, and future studies should attempt to assess dropouts across multiple waves to determine whether predictors attrition remain the same or differ across different intervals of time (eg, 4-months vs 1-year). Given the desire to understand participant’s dropping out early on in the data collection phase, this analysis focused on the first two waves. Although the analysis of dropout between all waves is recommended, the authors felt this initial approach would be very informative, given the current limitations in the literature.

### Conclusions and Future Directions

This study indicated that, in addition to individual and institutional factors previously explored in the literature, tobacco use at baseline predicted subsequent attrition at the follow-up assessment. Future studies should replicate these results in a broader sample of students and colleges not confined to one state. Although we obtained sufficient samples from each of our seven schools, replication of these findings in large samples and across other areas of the United States could potentially add to the knowledge of what predicts nonresponse in tobacco use studies in contextual settings. Additionally, this analysis highlights the need to understand both individual and contextual factors (including research topic and incentives for participation) that may have strong effects on decisions to continue participation in a survey study. Understanding these factors will allow for superior methods of tailoring recruiting efforts to those at highest risk of nonresponse and preventing bias due to systematic dropout in longitudinal studies. Moreover, these findings have implications for how such data are interpreted and also highlight the need to examine the impact of nonresponse over the course of such longitudinal studies. These efforts will assist researchers in decreasing study bias and developing best practices to decrease smoking and other substance use behaviors in college students, a vulnerable population to these behaviors.

## Abbreviations

DECOY: Documenting Experiences with Cigarettes and Other Tobacco in Young Adults
ICC: intraclass correlation coefficient
OR: odds ratio
